# Baicalin Down-Regulates IL-1β-Stimulated Extracellular Matrix Production in Nasal Fibroblasts

**DOI:** 10.1371/journal.pone.0168195

**Published:** 2016-12-21

**Authors:** Jae-Min Shin, Ju-Hyung Kang, Seoung-Ae Lee, Il-Ho Park, Heung-Man Lee

**Affiliations:** 1 Department of Otorhinolaryngology-Head and Neck Surgery, Korea University College of Medicine, Seoul, South Korea; 2 Division of Brain Korea 21 Program for Biomedical Science, Korea University College of Medicine, Seoul, South Korea; 3 Institute for Korea University Medical Devices Support Center, Korea University College of Medicine, Seoul, South Korea; Medical University of South Carolina, UNITED STATES

## Abstract

**Purpose:**

Baicalin, a Chinese herbal medicine, has anti-fibrotic and anti-inflammatory effects. The aims of present study were to investigate the effects of baicalin on the myofibroblast differentiation, extracellular matrix production, migration, and collagen contraction of interleukin (IL)-1β-stimulated nasal fibroblasts and to determine the molecular mechanism of baicalin in nasal fibroblasts.

**Methods:**

Nasal fibroblasts were isolated from the inferior turbinate of patients. Baicalin was used to treat IL-1β-stimulated nasal fibroblasts. To evaluate cytotoxicity, a 3-(4,5-dimethylthiazol-2yl)-2,5-diphenyl-tetrazolium bromide assay was used. The expression levels of α-smooth muscle actin (SMA), fibronectin, phospho-mitogen-activated protein kinase (p-MAPK), p-Akt, p-p50, p-p65, and p-IκBα were measured by western blotting, reverse transcription-polymerase chain reaction (RT—PCR),or immunofluorescence staining. Fibroblast migration was analyzed with scratch assays and transwell migration assays. Total collagen was evaluated with the Sircol collagen assay. Contractile activity was measured with a collagen gel contraction assay.

**Results:**

Baicalin (0–50 μM) had no significant cytotoxic effects in nasal fibroblasts. The expression of α*–*SMA and fibronectin were significantly down-regulated in baicalin-treated nasal fibroblasts. Migration, collagen production, and contraction of IL-1β-stimulated nasal fibroblasts were significantly inhibited by baicalin treatment. Baicalin also significantly down-regulated p-MAPK, p-Akt, p-p50, p-p65, and p-IκBα in IL-1β-stimulated nasal fibroblasts.

**Conclusions:**

We showed that baicalin down-regulated myofibroblast differentiation, extracellular matrix production, migration, and collagen contraction via the MAPK and Akt/ NF-κB pathways in IL-1β-stimulated nasal fibroblasts.

## Introduction

Outside environmental factors, such as microbes, pollutants, and allergens, first contact the respiratory immune system in the nose. As a result, upper airway inflammatory diseases are common. Chronic rhinosinusitis (CRS), an inflammatory disease of the nasal cavity and paranasal sinus, has a prevalence of 10% in developed countries [1]. Although the exact pathophysiologic mechanism of CRS has not been identified, chronic inflammation and airway remodeling characterized by extracellular matrix (ECM) deposition plays a key role in CRS development and propagation [2,3]. Fibroblasts have long been considered to provide structural tissue support and ECM secretion, but emerging evidence suggests that fibroblasts have a crucial role in chronic inflammation as an immune modulator that regulates the secretion and amplification of various inflammatory cytokines and chemokines [4]. According to recent studies, nasal fibroblasts are responsible for driving and contributing to upper airway inflammation [4].

Interleukin (IL)-1β, a member of the IL-1 family, is a pivotal pro-inflammatory cytokine deeply involved in chronic inflammation and tissue remodeling [5,6]. In particular, IL-1β significantly contributes to ECM accumulation and airway remodeling by interacting with transforming growth factor (TGF)-β in chronic inflammatory diseases of the lower airway such as asthma, chronic obstructive pulmonary disease, and idiopathic pulmonary fibrosis [6,7]. Moreover, evidence shows that IL-1β expression is significantly up-regulated in the nasal mucosa of patients with CRS, and, thus, it may be also associated with chronic inflammation of the upper airway [8].

Baicalin, a flavonoid compound extracted from the dried roots of *Scutellaria baicalensis* Georgi, has multiple pharmacological activities including anti-oxidant, anti-bacterial, anti-viral, and anti-inflammatory effects [9,10]. Recently, Liu *et al* showed that baicalin inhibits the synthesis and accumulation of collagen, a principal component of ECM, in a rat model of pulmonary hypertension [11]. Moreover, Sun *et al* demonstrated that baicalin significantly suppressed the effects of ovalbumin in a mouse model of asthmatic airway remodeling [12]. However, the effects and molecular mechanisms of baicalin in nasal fibroblasts are not yet known. The purpose of this study was to investigate the effects of baicalin on the myofibroblast differentiation, ECM production, migration, and collagen contraction of IL-1β-stimulated nasal fibroblasts and to determine the molecular mechanisms of baicalin in nasal fibroblasts.

## Materials and Methods

### Materials

Human recombinant IL-1β was obtained from Biovision (Milpitas, CA). Baicalin was provided by Sigma (St. Louis, MO). Inhibitors of extracellular signal-regulated kinase (ERK; U0126), p38 (SB203580), and c-Jun N-terminal kinase (JNK; SP600125) were purchased from Calbiochem (Billerica, MA). Inhibitors of Akt (LY294002) and NF-κB (BAY-117082) were provided by Sigma. Antibodies against phospho-ERK (p-ERK), total-ERK (t-ERK), p-p38, t-p38, p-JNK, t-JNK, p-Akt, t-Akt, p-p50, p50, p-p65, p65, p-IκBα, IκBα, fibronectin, α-smooth muscle actin (α-SMA), and β-actin were obtained from Santa Cruz Biotechnology (Santa Cruz, CA).

### Inferior turbinate-derived fibroblast culture

Patients were recruited from the Department of Otorhinolaryngology, Korea University Medical Center, Korea. Written informed consent was obtained from each patient, and the study was approved by the Korea University Medical Center Institutional Review Board. All patients provided their written informed consent to participate in this study and had no history of allergy, asthma, or aspirin sensitivity. Inferior turbinate-derived fibroblasts were isolated from six patients’ (three males and three females; mean age, 32.8 ± 7.9 years) surgical tissues by enzymatic digestion with collagenase (500 U/ml, Sigma), hyaluronidase (30 U/ml, Sigma), and DNase (10 U/ml, Sigma). Cells were cultured in Dulbecco's Modified Eagle Medium (DMEM) containing 10% (v/v) heat-inactivated fetal bovine serum (Invitrogen^TM^, Carlsbad, CA), 1% (v/v) 10,000 units/ml penicillin, and 10,000 μg/ml streptomycin (Invitrogen^TM^). According to our previous study, the purity of obtained cells was confirmed by characteristic spindle-shaped cell morphology and flow cytometry [3]. Cells were used in experiments from the fourth to seventh cell passages.

### 3-(4,5-dimethylthiazol-2-yl)-2,5-diphenyl tetrazolium bromide assay

To determine the cytotoxic effects of baicalin in nasal fibroblasts, an MTT (3-(4,5-dimethylthiazol-2-yl)-2,5-diphenyl tetrazolium bromide, Sigma) assay was used. Fibroblasts were treated with baicalin (0–400 μM) for 72hours. And then, incubated with MTT for 4 hours, and the reaction was interrupted by adding dimethyl sulfoxide. A fluorescence microplate reader (F2000; Hitachi, Ltd., Tokyo, Japan) was used at 570 nm to measure the results.

### Semiquantitative reverse transcription-polymerase chain reaction (RT-PCR)

Nasal fibroblasts were pre-treated with baicalin (0–50 μM) for 1 hour, and then stimulated with IL-1β (10 ng/ml) for 24 hours. Total RNA was isolated with the Trizol reagent (Invitrogen^TM^). Two micrograms of RNA was reverse-transcribed with Moloney murine leukemia virus reverse transcriptase (Invitrogen^TM^). Polymerase chain reaction (PCR) was performed with the following primers: *α-SMA* (sense 5′ - GGT GCT GTC TCT CTA GCC TCT GGA—3′, anti-sense 5′ - CCC ATC AGG CAA CTC GAT ACT CTT C—3′, 322 bp), *fibronectin* (sense 5’—GGA TGC TCC TGC TGT CAC - 3’, anti-sense 5’- CTG TTT GAT CTG GAC CTG CAG—3’, 386 bp), *collagen type I* (sense 5′- CAT CAC CTA CCA CTG CAA GAA C—3′, anti-sense 5′- ACG TCG AAG CCG AAT TCC—3′, 278 bp), and *GAPDH* (sense 5’- GTG GAT ATT GTT GCC ATC AAT GAC C—3’, anti-sense 5’- GCC CCA GCC TTC TTC ATG GTG GT—3’, 271 bp). Amplification reactions were performed as follows: an initial 5-minute denaturation step at 94°C; followed by 30 cycles at 94°C for 45 seconds, 55–65°C for 45 seconds, and 72°C for 45 seconds; and a final extension step at 74°C for 5 minutes. All reactions were performed in a 20 μl volume. Products were electrophoresed on a 1.5% agarose gel and visualized by staining with ethidium bromide. The gels were captured and visualized with a Molecular Imager ChemiDoc XRS+ (Bio-Rad, Hercules, CA).

### Western blot analysis

For detection of α-SMA, fibronectin and β-actin, nasal fibroblasts were pre-treated for 1 hour with the following baicalin (50 μM) and specific inhibitors: U0126 (ERK inhibitor, 10 μM), SB203580 (p38 inhibitor, 10 μM), SP600125 (JNK inhibitor, 10 μM), LY294002 (AKT inhibitor, 10 μM), and BAY-117082 (NF-κB inhibitor, 1 μM). After then, cells were stimulated IL-1β (10 ng/ml) for 72 hours. For detection of p-ERK, p-p38, p-JNK, p-Akt and p-IκBα, nasal fibroblasts were pre-treated for 1 hour with the following baicalin and specific inhibitors: U0126, SB203580, SP600125, LY294002, BAY-117082. And then, cells were stimulated IL-1β for various times (0–120 minutes). Cells were lysed in PRO-PREP^TM^ protein extraction solution (iNtRON Biotechnology, Seongnam, Korea). Lysates were separated by 10% sodium dodecyl sulfate-polyacrylamide gel electrophoresis and transferred onto polyvinyl difluoride membranes (Millipore Inc., Billerica, MA). Membranes were blocked with 5% skim milk solution and incubated with the following antibodies: α-SMA, fibronectin, p-ERK, t-ERK, p-p38, t-p38, p-JNK, t-JNK, p-Akt, t-Akt, p-p50, p50, p-p65, p65, p-IκBα, IκBα and β-actin. Blots were visualized with HRP-conjugated secondary antibodies and an ECL system (Pierce, Rockford, IL).

### Immunofluorescence staining

Nasal fibroblasts on coverslips were pre-treated for 1 hour with the baicalin (50 μM), and then, stimulated with IL-1β (10 ng/ml) for 72 hours. Cells were fixed with 4% paraformaldehyde, permeabilized with 0.2% Triton X-100 in 1% bovine serum albumin for 10 minutes, blocked with 5% bovine serum albumin for 1 hour at room temperature, and incubated overnight at 4°C with monoclonal anti-α-SMA or polyclonal anti-fibronectin. Fibroblasts were then incubated with anti-mouse Alexa Fluor 488 (Invitrogen^TM^) or anti-rabbit Alexa Fluor 555 (Invitrogen^TM^) secondary antibodies. Finally, coverslips were counterstained with 4′-6-diamidino-2-phenylindole. Stained fibroblasts were captured and visualized under a confocal laser scanning microscope (LSM700, Zeiss, Oberkochen, Germany).

### Cell migration scratch assays

Nasal fibroblasts were plated and grown to confluence in 6-well tissue culture dishes. A straight scratch was made in the cells using a pipette tip. Scratched cells were immediately rinsed with phosphate buffered saline and DMEM medium containing 10% (v/v) heat-inactivated fetal bovine serum (Invitrogen^TM^), 1,000 unit/ml penicillin, and 1,000 μg/ml streptomycin (Invitrogen^TM^) was added. Cells were incubated with IL-1β (10 ng/ml) alone or in conjunction with baicalin (50 μM) up to 48 hours. Images were obtained with a microscope (Olympus BX51; Olympus, Tokyo, Japan. The cell migration rate was expressed as the ratio of the migration distance in control cells (100%).

### Transwell migration assay

Fibroblasts were seeded in the upper chamber of transwell chambers (Corning Life Sciences, MA). Then, DMEM containing 10% (v/v) heat-inactivated fetal bovine serum (Invitrogen^TM^), 1,000 unit/ml penicillin, and 1,000 μg/ml streptomycin (Invitrogen^TM^) was added with IL-1β (10 ng/ml) alone or in conjunction with baicalin (50 μM) to the lower chamber up to 48 hours. The cells on the upper surface of the membrane were removed with cotton swabs. Cells on the lower surface of the membrane were stained with Diff-Quik stain (Sysmex, Kobe, Japan). Images of the stained cells from five selected views were captured with a microscope at 200x magnification at 0, 48 hours.

### Collagen measurements

Nasal fibroblasts were pre-treated for 1 hour with the following baicalin (50 μM) and specific inhibitors: U0126 (ERK inhibitor, 10 μM), SB203580 (p38 inhibitor, 10 μM), SP600125 (JNK inhibitor, 10 μM), LY294002 (AKT inhibitor, 10 μM), and BAY-117082 (NF-κB inhibitor, 1 μM). After then, cells were stimulated with IL-1β (10 ng/ml) for 72 hours. As previously reported [13], total soluble collagen in cell culture supernatants was quantified using the Sircol collagen assay (Biocolor, Belfast, UK). One milliliter of Sirius red dye, an anionic dye that reacts specifically with basic side chain groups on collagen under the assay conditions, was added to 400 μl of supernatant and incubated with gentle rotation for 30 minutes at room temperature. The samples were centrifuged at 12,000 rpm for 10 minutes, and the collagen–dye complex precipitate was collected and re-solubilized in 0.5 M sodium hydroxide. The dye concentration was estimated by spectrophotometry at 540 nm (Beckman Coulter, Fullerton, CA). The absorbance was directly proportional to the amount of newly formed soluble collagen in the cell culture supernatant.

### Collagen gel contraction assay

Rat-tail tendon collagen type I was purchased from BD Biosciences (Bedford, MA). Collagen gels were prepared as previously described by mixing rat-tail tendon collagen type I, serum-free DMEM, and cells. Fibroblasts were then mixed with the neutralized collagen solution (pH 7.4) so that the final cell density in the collagen solution was 3 x 10^5^ cells/ml, and the final collagen concentration was 0.75 mg/ml. Aliquots (0.5 ml/well) of the mixture of cells in collagen were cast into each well of 24-well tissue culture plates and allowed to polymerize at room temperature, generally completed in 20 minutes. After polymerization, the gels were gently released from the 24-well tissue culture plates and transferred to 6-well culture plates containing 1.5 ml serum free-DMEM with IL-1β, baicalin, or both. The gels were then incubated at 37°C in a 5% CO_2_ atmosphere for 3 days. The area of each gel was measured with Image J analyzer (National Institutes of Health, Bethesda, MD). Data are expressed as a percentage of the initial gel area.

### Collagen zymography

Nasal fibroblasts were pre-treated for 1 hour with the baicalin (50 μM), and then, stimulated with IL-1β (10 ng/ml) for 72 hours. Aliquots of medium conditioned (10 μl) by cells were analyzed using collagen zymography for matrix-metalloproteinase (MMP)-1 in 0.4 mg/mL collagen-10% polyacrylamide gels. Following electrophoresis, the gels were washed twice with 2.5% Triton X-100 for 30 minutes while shaking to remove sodium dodecyl sulfate-polyacrylamide and to renature the MMP-1 in the gels. Renaturated gels were incubated in developing buffer containing 100 mM Tris–HCl, 5 mM CaCl2, 0.005% Brij-35, and 0.001% NaN3 (pH 8.0), overnight at 37°C. Gels were stained with 0.25% Coomassie brilliant blue G-250 (50% methanol, 10% acetic acid) and destained using destaining solution (50% methanol, 10% acetic acid). Proteinase activity was observed as cleared (unstained) regions. Finally, the gels were dried for 2 hours using a gel dryer (Bio-Rad Labs).

### Statistical analysis

Results were obtained from at least three independent experiments. Statistically significant differences between control and experimental data were analyzed with unpaired *t* test or one-way analysis of variance followed by Tukey’s test (GraphPad Prism, version 5, Graph Pad Software, San Diego, CA). Significance was established at the 95% confidence level. *P* values less than 0.05 were accepted as statistically significant.

## Results

### Baicalin inhibits IL-1β-stimulated myofibroblast differentiation (α-SMA) and ECM production in nasal fibroblasts

**Fig 1:**
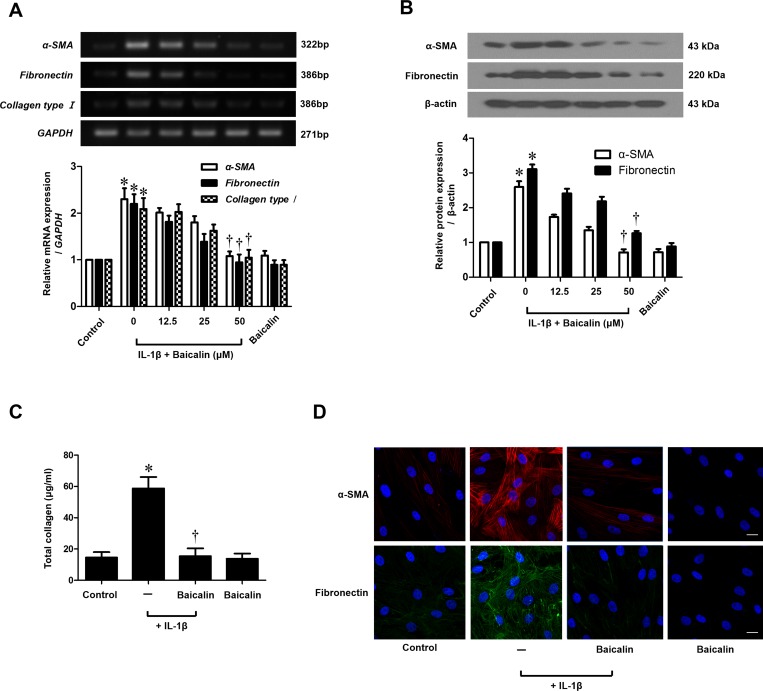
Effect of baicalin on IL-1β-stimulated myofibroblast differentiation and extracellular matrix production in nasal fibroblasts. (A) After treatement with IL-1β (10 ng/ml) with or without baicalin (0–50 μM) for 24 hours, expression level of *α-SMA*, *fibronectin* and *collagen type I* mRNA was determined by RT—PCR. (B) After treatement with IL-1β (10 ng/ml) with or without baicalin (0–50 μM) for 72 hours, level of α-SMA and fibronectin protein was measured by western blot and (C) the total amount of collagen was measured by Sircol assay. (D) After treatement with IL-1β (10 ng/ml) with or without baicalin (0–50 μM) for 72 hours, localization of α-SMA and fibronectin protein were observed by immunocytochemical staining. Values are the means ± SEM of three independent experiments. Images were acquired by confocal laser scanning microscopy. *p < 0.05 vs. control; ^†^*p* < 0.05 vs. IL-1β alone. Scale bar = 50 μm.

To determine the cytotoxic effects of baicalin on nasal fibroblasts, an MTT (3-(4,5-dimethylthiazol-2-yl)-2,5-diphenyl tetrazolium bromide, Sigma) assay was used. Baicalin was not cytotoxic for 72 hours at concentrations up to 50 μM (S1 Fig). To examine the inhibitory effects of baicalin on α-SMA, fibronectin and collagen accumulation in nasal fibroblasts, cells were treated with IL-1β with or without baicalin at different concentrations (0–50 μM). Baicalin significantly down-regulated expression of IL-1β-induced α-SMA, fibronectin and collagen type I mRNA, as assessed by RT—PCR (*p* < 0.05, Fig 1A). IL-1β-induced expression of α-SMA and fibronectin protein was suppressed by baicalin (Fig 1B). Expression levels of collagen protein were determined with a Sircol soluble collagen assay. IL-1β-stimulated expression of collagen protein was significantly down-regulated by baicalin treatment (*p* < 0.05, Fig 1C). Localization of α-SMA and fibronectin was determined by immunocytochemical staining (Fig 1D). Expressions of α-SMA and fibronectin protein were markedly down-regulated by baicalin in a dose-dependent manner. These results indicate that IL-1β-stimulated myofibroblast differentiation and ECM production were significantly inhibited by baicalin.

### Baicalin inhibits IL-1β–stimulated migration in nasal fibroblasts

**Fig 2:**
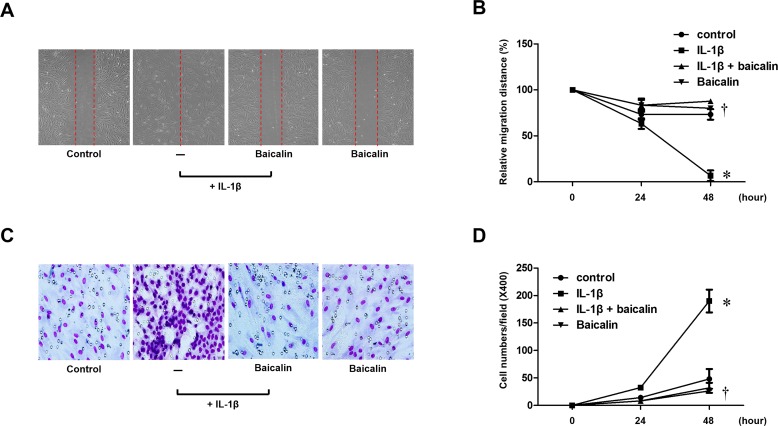
Effect of baicalin on IL-1β-stimulated migration in nasal fibroblasts. Nasal fibroblasts were stimulated with IL-1β (10 ng/ml) alone or in conjunction with baicalin (50 μM) up to 48 hours. (A) Phase-contrast images (200x) of the migration scratch assay at 48 hours. (B) The distance of cell migration was measured from the phase-contrast images (200x) taken at 0 to 48 hours. (C) Phase-contrast images (200x) of the transwell migration assay at 48 hours. (D) The number of cell migration was counted from phase-contrast images (400x) taken at 0 to 48 hours. Values are the mean ± SEM of independent samples. *p < 0.05 vs. control; ^†^*p* < 0.05 vs. IL-1β alone.

To evaluate the effects of baicalin on IL-1β-stimulated migration of nasal fibroblasts, scratch and transwell migration assays of cell migration were performed. Nasal fibroblasts were treated with mitomycin C to inhibit cell proliferation. Then, fibroblasts were treated with IL-1β with or without baicalin. The migration distances were significantly higher in cells treated with IL-1β alone compared with cells treated with IL-1β and baicalin (*p* < 0.05, Fig 2A and 2B). In addition, baicalin also significantly suppressed IL-1β-stimulated migration in a transwell migration assay (*p* < 0.05, Fig 2C and 2D). Taken together, these results suggest that baicalin inhibits IL-1β-stimulated migration of nasal fibroblasts.

### Baicalin inhibits IL-1β-stimulated collagen contraction and activity of MMP-1 in nasal fibroblasts

**Fig 3:**
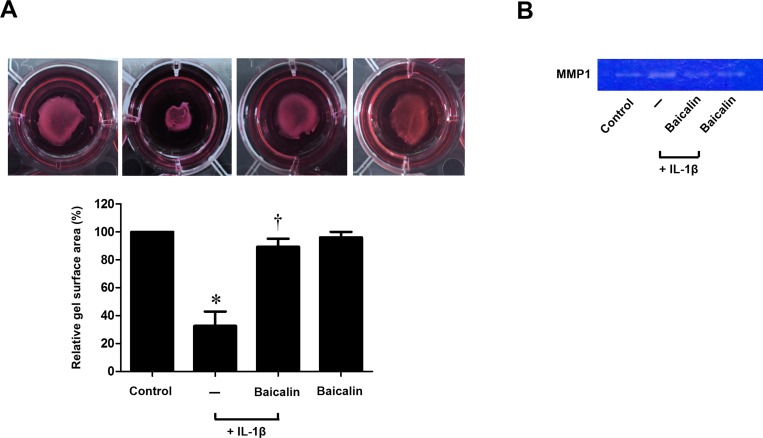
Effect of baicalin on IL-1β-stimulated collagen contraction and activity of MMP-1 in nasal fibroblasts. Nasal fibroblasts were stimulated with IL-1β (10 ng/ml) alone or in conjunction with baicalin (50 μM) for 72 hours. (A) Contractile activity was assessed by a collagen gel contraction assay and the contraction area was measured. (B) Collagenase activity, matrix-metalloproteinase (MMP)-1 secretion was measured using collagen zymography. Values are the mean ± SEM of independent samples. *p < 0.05 vs. control; ^†^*p* < 0.05 vs. IL-1β alone.

To investigate the effect of baicalin on collagen contraction and activity of MMP-1, fibroblasts were treated with IL-1β with or without baicalin. Baicalin significantly inhibited IL-1β-stimulated contraction as assessed by a collagen gel contraction assay (*p* < 0.05, Fig 3A). MMP-1 enzymatic activity was analyzed by zymography. IL-1β-stimulated expression of MMP-1 was significantly down-regulated by baicalin treatment (*p* < 0.05, Fig 3B). These results indicate that baicalin inhibits IL-1β-stimulated collagen contraction and activity of MMP-1 in nasal fibroblasts.

### Mitogen-activated protein kinase (MAPK) and Akt regulate IL-1β-stimulated myofibroblast differentiation and ECM production in nasal fibroblasts

**Fig 4:**
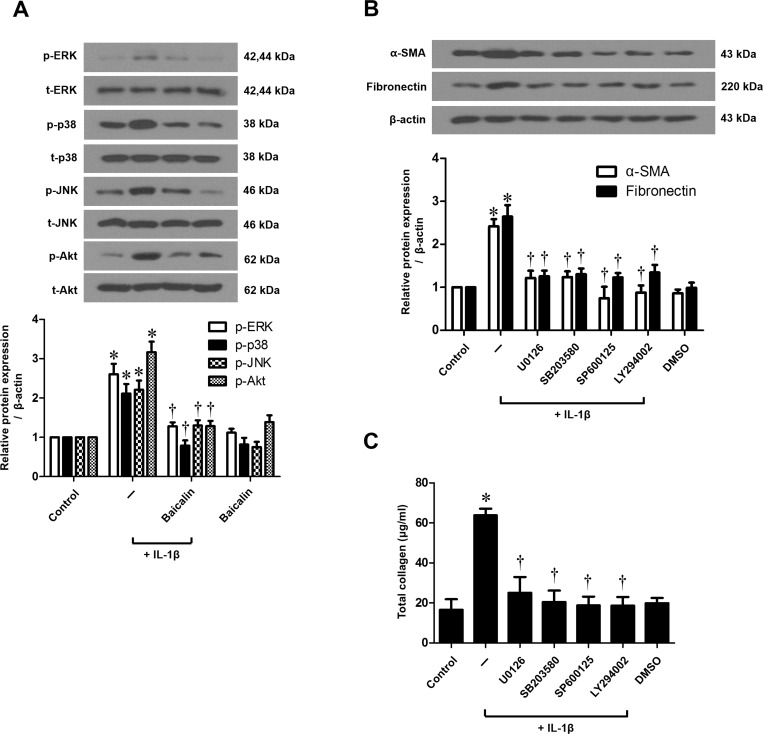
Effect of baicalin on IL-1β-stimulated signaling pathways in nasal fibroblasts. (A) Nasal fibroblasts were stimulated with IL-1β (10 ng/ml) alone or in conjunction with baicalin (50 μM) for 30 minutes. The phosphorylation of signaling molecules including p-ERK, t-ERK, p-p38, t-p38, p-JNK, t-JNK, p-Akt and t-Akt was measured by western blotting. (B) Nasal fibroblasts were stimulated with IL-1β (10 ng/ml), with or without baicalin (50 μM) and mitogen-activated protein kinase (MAPK) inhibitors, including extracellular signal-regulated kinase (ERK; U0126, 10 μM), p38 (SB203580, 10 μM), and c-Jun N-terminal kinase (JNK; SP600125, 10 μM) inhibitors, and an AKT inhibitor (LY294002, 10 μM) for 72 hours. The expression level of α-SMA and fibronectin protein was determined by western blotting and (C) total collagen was measured by the Sircol assay. Values are the means ± SEM of three independent experiments. *p < 0.05 vs. control; ^†^*p* < 0.05 vs. IL-1β alone.

To verify whether the MAPK (ERK, p38, and JNK) and Akt signaling pathway is involved in α-SMA, fibronectin, and collagen expressions, fibroblasts were treated with IL-1β with or without baicalin and MAPK or Akt inhibitors. We examined the activation of MAPK and Akt signal proteins by western blot and determined that IL-1β activated all three MAPKs (ERK, p38, and JNK) and Akt. Baicalin markedly down-regulated the phosphorylation of the three MAPKs and Akt (Fig 4A). In addition, IL-1β-stimulated expression of α-SMA, fibronectin, and collagen were significantly down-regulated by all three MAPK inhibitors and the Akt inhibitor (*p* < 0.05, Fig 4B and 4C). These results indicate that baicalin regulates IL-1β-stimulated myofibroblast differentiation and ECM production via MAPK and Akt signaling pathways in nasal fibroblasts.

### NF-κB regulates IL-1β-stimulated myofibroblast differentiation and ECM production in nasal fibroblasts

**Fig 5:**
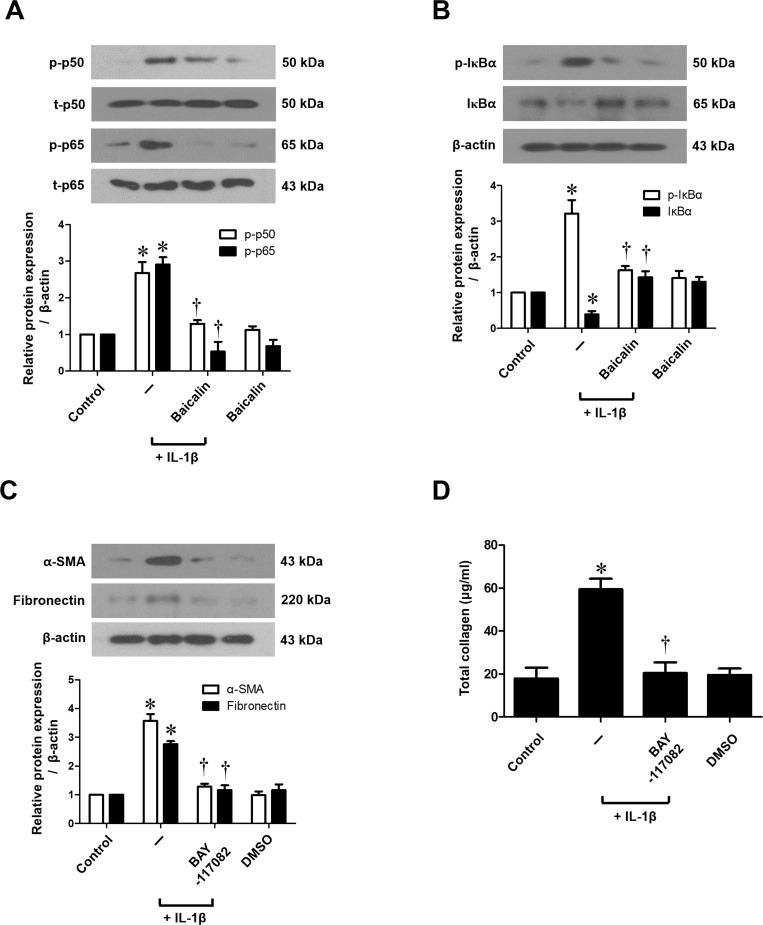
Effect of baicalin on IL-1β-stimulated NF-κB signaling in nasal fibroblasts. (A) Nasal fibroblasts were stimulated with IL-1β (10 ng/ml) alone or in conjunction with baicalin (50 μM) for 60 minutes. p-p50, p50, p-p65 and p65 protein production were determined by western blotting and (B) protein expression of p-IκBα and IκBα were assessed by western blotting. (C) Nasal fibroblasts were stimulated with IL-1β (10 ng/ml), with or without baicalin (50 μM) or an NF-κB inhibitor (BAY-117082, 1 μM) for 72 hours. α-SMA and fibronectin protein expression was examined by western blotting and (D) total collagen was measured by the Sircol assay. Values are the means ± SEM of three independent experiments. *p < 0.05 vs. control; ^†^*p* < 0.05 vs. IL-1β alone.

To determine the role of NF-κB signaling in the expression of α-SMA, fibronectin and collagen, fibroblasts were treated with IL-1β, with or without baicalin or an NF-κB inhibitor (BAY-117082). Expressions of p-p50 and p-p65 protein, the active forms of NF-κB subunits, were up-regulated by IL-1β, and then markedly inhibited by baicalin treatment (Fig 5A). Moreover, the treatment of IL-1β showed significantly greater phosphorylation of IκBα (NF-κB inhibitor) than IL-1β-untreated nasal fibroblasts, while baicalin significantly down-regulated (Fig 5B). Further, the NF-κB inhibitor (BAY-117082) significantly down-regulated IL-1β-stimulated production of α-SMA, fibronectin, and collagen (*p* < 0.05, Fig 5C and 5D). These results suggest that the effect of baicalin on IL-1β-stimulated myofibroblast differentiation and ECM production may be mediated by NF-κB pathways in nasal fibroblasts.

## Discussion

The present study demonstrated that baicalin down-regulates IL-1β-stimulated myofibroblast differentiation (*α*-SMA), ECM production (fibronectin and collagen), migration, and collagen contraction, which is related to suppression of the MAPK and Akt/NF-κB signaling pathway in nasal fibroblasts.

IL-1β is involved in chronic inflammation, ECM metabolism, and tissue remodeling in a variety of tissues and organs [14,15]. In present study, we clearly showed that IL-1β induced myofibroblast differentiation, ECM production, migration, and collagen contraction in nasal fibroblasts. Furthermore, White *et al*. demonstrated that increased IL-1β during acute lung injury induced lung fibroblast proliferation and collagen production [16]. These results agree with previous studies that reported that IL-1β induces an increase in collagen synthesis and secretion in human dental and gingival fibroblasts [17,18]. Conversely, our results differ from those of Xiao *et al*. who found that IL-1β inhibited collagen synthesis and increased MMP activity in cultured cardiac fibroblasts [19]. Therefore, controversy remains regarding the direct effect of IL-1β on fibroblasts. Furuyama *et al*. suggest a dual action in which IL-1β increased ECM accumulation and contributed to basement membrane formation and remodeling in rat pulmonary fibroblasts, while IL-1β also contributed to ECM degradation through MMP-2 and MMP-9 secretion [20]. This discrepancy in the effect of IL-1β may be related to fibroblasts of different origins and differences in IL-1β concentration and exposure time.

NF-κB, the transcription factor, plays a significant role in cellular response for inflammation, infection and immunity. It was retained in an inactive form after combining with the inhibitory protein (IκB) inside of cytoplasm [21]. Various stimulations lead phosphorylation and subsequently proteasome-mediated degradation of IκB [22]. Then, the free-subunits of NF-κB are phosphorylated and translocated into nucleus activating transcription of target genes [23]. In the current study, we consistently shown that Baicalin inhibits IL-1β-induced phosphorylation of p50, p65 and IκBα in nasal fibroblast. Further, we demonstrated that NF-κB specific inhibitor (Bay-117082) suppresses IL-1β-stimulated myofibroblast differentiation (a-SMA) and ECM production (fibronectin and collagen) in nasal fibroblast. Taken together, these results suggest that the effect of baicalin on IL-1β-stimulated myofibroblast differentiation and ECM production may be mediated by NF-κB pathways in nasal fibroblasts.

Hu *et al* reported that among the various traditional Chinese herbal medicines that suppress ECM accumulation, baicalin significantly inhibited total collagen deposition and had relatively low cytotoxicity in an in vitro model of renal fibrosis [24]. Accordingly, we focused on baicalin and hypothesized that it suppresses the ECM deposition in upper airway inflammatory diseases. In our study, we clearly demonstrated that baicalin down-regulated myofibroblast differentiation, ECM production, migration, and collagen contraction in IL-1β-induced nasal fibroblasts. These results suggest that baicalin may attenuate upper airway remodeling. These results are consistent with those of previous studies that reported that baicalin inhibited IL-1β secretion and has therapeutic effects on collagen-induced inflammation in human fibroblast-like synoviocytes and a rat model of collagen-induced arthritis [25].

To our knowledge, this is the first study to describe the roles of baicalin and the underlying mechanism of IL-1β-stimulated myofibroblast differentiation, ECM production, migration, and collagen contraction in nasal fibroblasts. Baicalin (0–50 μM) had no significant cytotoxic effects in nasal fibroblasts, and its effects on myofibroblast differentiation and ECM production were dose dependent.

In summary, our results show that baicalin down-regulates IL-1β-stimulated myofibroblast differentiation, ECM production, migration, and collagen contraction via the MAPK and Akt/NF-κB signaling pathways in nasal fibroblasts. Therefore, baicalin may provide a potential therapeutic option for chronic upper airway inflammatory disease such as CRS.

## Supporting Information

S1 Fig Cytotoxicity of baicalin in nasal fibroblasts. Cytotoxicity was assessed with a 2-(4,5-dimethylthiazol-2yl)-2,5-diphenyl-2H-tetrazolium bromide (MTT) assay at various baicalin concentrations in nasal fibroblasts. Values are the means ± SEM of three independent samples.
(TIF)
