# Supplementary figures and images for: Baicalin Down-Regulates IL-1β-Stimulated Extracellular Matrix Production in Nasal Fibroblasts
Source: PLoS One. 2016 Dec 21;11(12):e0168195. doi: 10.1371/journal.pone.0168195 (PMC5176301; doi:10.1371/journal.pone.0168195)

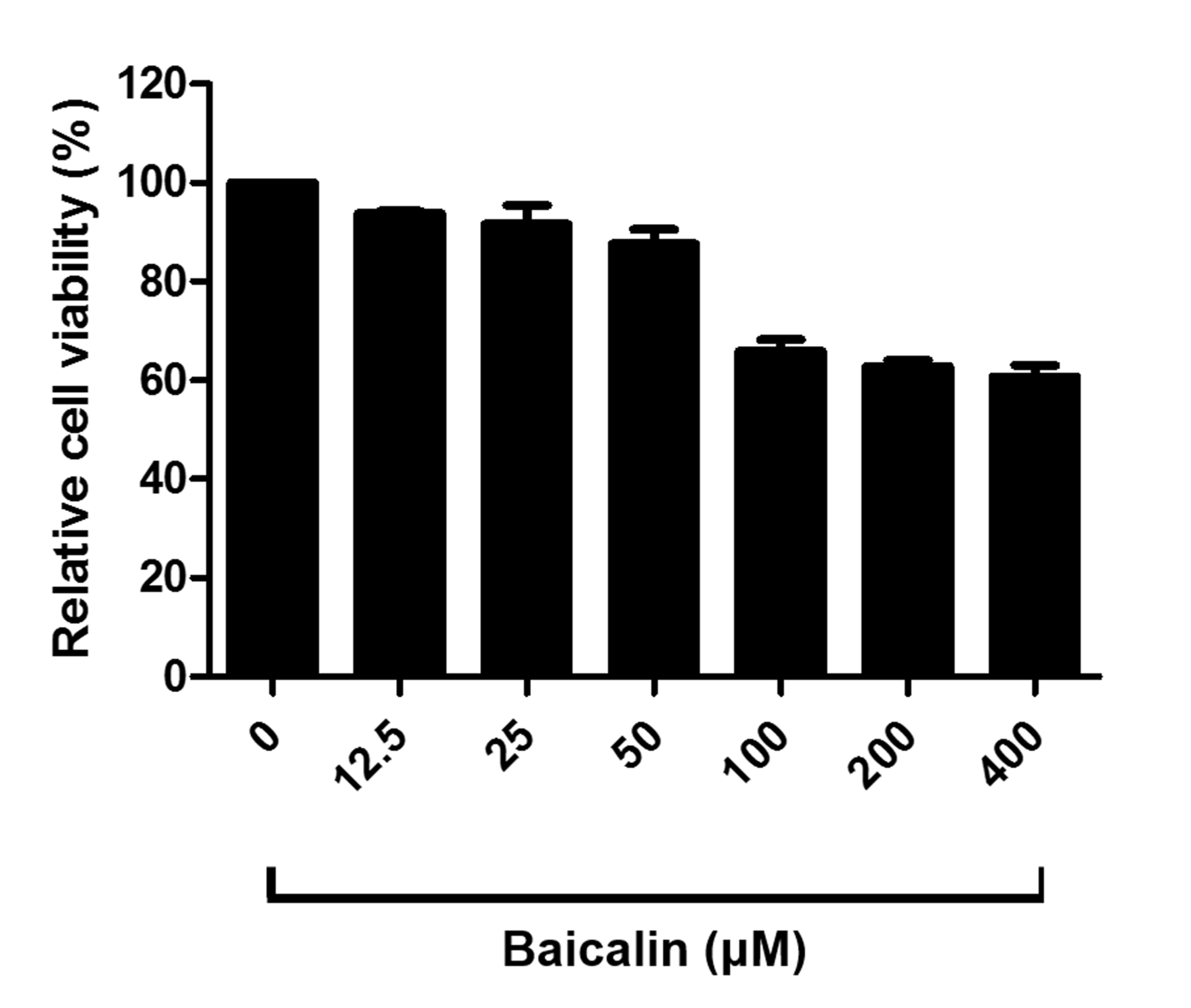

Supplement: S1 Fig — Cytotoxicity was assessed with a 2-(4,5-dimethylthiazol-2yl)-2,5-diphenyl-2H-tetrazolium bromide (MTT) assay at various baicalin concentrations in nasal fibroblasts. Values are the means ± SEM of three independent samples. (TIF) [file pone.0168195.s001.tif]
